# Development and external evaluation of a self-learning auto-segmentation model for Colorectal Cancer Liver Metastases Assessment (COALA)

**DOI:** 10.1186/s13244-024-01820-7

**Published:** 2024-11-22

**Authors:** Jacqueline I. Bereska, Michiel Zeeuw, Luuk Wagenaar, Håvard Bjørke Jenssen, Nina J. Wesdorp, Delanie van der Meulen, Leonard F. Bereska, Efstratios Gavves, Boris V. Janssen, Marc G. Besselink, Henk A. Marquering, Jan-Hein T. M. van Waesberghe, Davit L. Aghayan, Egidijus Pelanis, Janneke van den Bergh, Irene I. M. Nota, Shira Moos, Gunter Kemmerich, Trygve Syversveen, Finn Kristian Kolrud, Joost Huiskens, Rutger-Jan Swijnenburg, Cornelis J. A. Punt, Jaap Stoker, Bjørn Edwin, Åsmund A. Fretland, Geert Kazemier, Inez M. Verpalen, for the Pancreatobiliary and Hepatic Artificial Intelligence Research (PHAIR) consortium, Giovanni Marchegiani, Domenico Bassi, Riccardo Boetto, Mattia Ballo, Riccardo Carandina, Filippo Crimi, Matteo Fassan, Arantza Farina, Caroline Verbeke, Knut Jørgen Labori, Åsmund Fretland, Mirko D’Onofrio, Giulia Zamboni, Riccardo di Robertis, Claudio Luchini, Alberto Balduzzi, Giuseppe Malleo, Roberto Salvia, Christopher Wolfgang, Ammar Javed, Katie Colborn, Marco Del Chiaro, Jeffrey Kaplan, Toshimasa Clark, Thomas Stoop, Ioana Lupescu, Cristian Mugur Grasu, Cristian Anghel, Mihai Dan Pomohaci, Philipp Mayer, Benedict Kinny-Köster, Martin Loos, Christoph Michalski, the Dutch Colorectal Cancer Group Liver Expert Panel, Martinus J. van Amerongen, Marinde J. G. Bond, Thiery Chapelle, Ronald M. van Dam, Marc R. W. Engelbrecht, Michael F. Gerhards, Dirk J. Grunhagen, Thomas M. van Gulik, John J. Hermans, Koert P. de Jong, Joost M. Klaase, Niels F. M. Kok, Wouter K. G. Leclercq, Mike S. L. Liem, Krijn P. van Lienden, I. Quintus Molenaar, Gijs A. Patijn, Arjen M. Rijken, Theo M. Ruers, Cornelis Verhoef, Johannes H. W. de Wilt

**Affiliations:** 1 Cancer Center Amsterdam, Amsterdam, The Netherlands; 2 Amsterdam UMC, University of Amsterdam, Department of Radiology and Nuclear Medicine, Amsterdam, The Netherlands; 3 Amsterdam UMC, University of Amsterdam, Department of Biomedical Engineering and Physics, Amsterdam, The Netherlands; 4 Amsterdam UMC, Vrije Universiteit Amsterdam, Department of Surgery, Amsterdam, The Netherlands; 5 Oslo University Hospital, Department of Radiology and Nuclear Medicine, Oslo, Norway; 6 University of Amsterdam, Video and Image Sense Lab, Amsterdam, The Netherlands; 7 Amsterdam Gastroenterology Endocrinology and Metabolism, Amsterdam, The Netherlands; 8 Amsterdam UMC, University of Amsterdam, Department of Surgery, Amsterdam, The Netherlands; 9 Amsterdam UMC, Vrije Universiteit Amsterdam, Department of Radiology and Nuclear Medicine, Amsterdam, The Netherlands; 10 Oslo University Hospital, Department of Hepato-Pancreato-Biliary Surgery, Oslo, Norway; 11 Oslo University Hospital, The Intervention Centre, Oslo, Norway; 12 Amsterdam UMC, University of Amsterdam, Department of Medical Oncology, Amsterdam, The Netherlands; 13 Department of Epidemiology, Julius Center for Health Sciences and Primary Care, University Medical Center Utrecht, Utrecht, The Netherlands; 14 Department of Surgery, Padova University Hospital, Padova, Italy; 15 Department of Radiology, Padova University Hospital, Padova, Italy; 16 Department of Pathology, University of Padova, Padova, Italy; 17 Department of Pathology, Amsterdam University Medical Centre, Amsterdam, The Netherlands; 18 Department of Pathology, Oslo University Hospital, Oslo, Norway; 19 Department of Surgery, Oslo University Hospital, Oslo, Norway; 20 Department of Radiology, Verona University Hospital, Verona, Italy; 21 Department of Pathology, University of Verona, Verona, Italy; 22 Department of Surgery, Verona University Hospital, Verona, Italy; 23 Department of Surgery, NYU Langone Health, New York, NY USA; 24 Department of Surgery, University of Colorado Anschutz Medical Campus, Aurora, CO USA; 25 Department of Pathology, University of Colorado Anschutz Medical Campus, Aurora, CO USA; 26 Department of Radiology, University of Colorado Anschutz Medical Campus, Aurora, CO USA; 27 Department of Radiology, Bucharest University Emergency Hospital, Bucharest, Romania; 28 Department of Radiology, Heidelberg University Hospital, Heidelberg, Germany; 29 Department of Surgery, Heidelberg University Hospital, Heidelberg, Germany; 30 Department of Radiology, Sint Maartenskliniek, Nijmegen, The Netherlands; 31 Department of Hepatobiliary, Transplantation, and Endocrine Surgery, Antwerp University Hospital, Antwerp, Belgium; 32 Department of Surgery, Maastricht University Medical Centre, Maastricht, The Netherlands; 33 Department of Surgery, OLVG Hospital, Amsterdam, The Netherlands; 34 Department of Surgical Oncology and Gastrointestinal Surgery, Erasmus MC Cancer Institute, Rotterdam, The Netherlands; 35 Department of Medical Imaging, Radboud University Medical Center, Radboud University Nijmegen, Nijmegen, The Netherlands; 36 Department of HPB Surgery and Liver Transplantation, University of Groningen, University Medical Center Groningen, Groningen, The Netherlands; 37 Department of Surgery, Netherlands Cancer Institute, Amsterdam, The Netherlands; 38 Department of Surgery, Máxima Medical Center, Veldhoven, The Netherlands; 39 Department of Surgery, Medical Spectrum Twente, Enschede, The Netherlands; 40 Department of Interventional Radiology, St Antonius Hospital, Nieuwegein, The Netherlands; 41 Department of Surgery, Regional Academic Cancer Center Utrecht, University Medical Center Utrecht and St Antonius Hospital, Nieuwegein, The Netherlands; 42 Department of Surgery, Isala Hospital, Zwolle, The Netherlands; 43 Department of Surgery, Amphia Hospital, Breda, The Netherlands; 44 Department of Surgery, Radboud University Medical Center, Radboud University Nijmegen, Nijmegen, The Netherlands

**Keywords:** Colorectal neoplasms, Liver, Biomarkers, Tumor, Artificial intelligence

## Abstract

**Objectives:**

Total tumor volume (TTV) is associated with overall and recurrence-free survival in patients with colorectal cancer liver metastases (CRLM). However, the labor-intensive nature of such manual assessments has hampered the clinical adoption of TTV as an imaging biomarker. This study aimed to develop and externally evaluate a CRLM auto-segmentation model on CT scans, to facilitate the clinical adoption of TTV.

**Methods:**

We developed an auto-segmentation model to segment CRLM using 783 contrast-enhanced portal venous phase CTs (CT-PVP) of 373 patients. We used a self-learning setup whereby we first trained a teacher model on 99 manually segmented CT-PVPs from three radiologists. The teacher model was then used to segment CRLM in the remaining 663 CT-PVPs for training the student model. We used the DICE score and the intraclass correlation coefficient (ICC) to compare the student model’s segmentations and the TTV obtained from these segmentations to those obtained from the merged segmentations. We evaluated the student model in an external test set of 50 CT-PVPs from 35 patients from the Oslo University Hospital and an internal test set of 21 CT-PVPs from 10 patients from the Amsterdam University Medical Centers.

**Results:**

The model reached a mean DICE score of 0.85 (IQR: 0.05) and 0.83 (IQR: 0.10) on the internal and external test sets, respectively. The ICC between the segmented volumes from the student model and from the merged segmentations was 0.97 on both test sets.

**Conclusion:**

The developed colorectal cancer liver metastases auto-segmentation model achieved a high DICE score and near-perfect agreement for assessing TTV.

**Critical relevance statement:**

AI model segments colorectal liver metastases on CT with high performance on two test sets. Accurate segmentation of colorectal liver metastases could facilitate the clinical adoption of total tumor volume as an imaging biomarker for prognosis and treatment response monitoring.

**Key Points:**

- Developed colorectal liver metastases segmentation model to facilitate total tumor volume assessment.
- Model achieved high performance on internal and external test sets.
- Model can improve prognostic stratification and treatment planning for colorectal liver metastases.

**Graphical Abstract:**

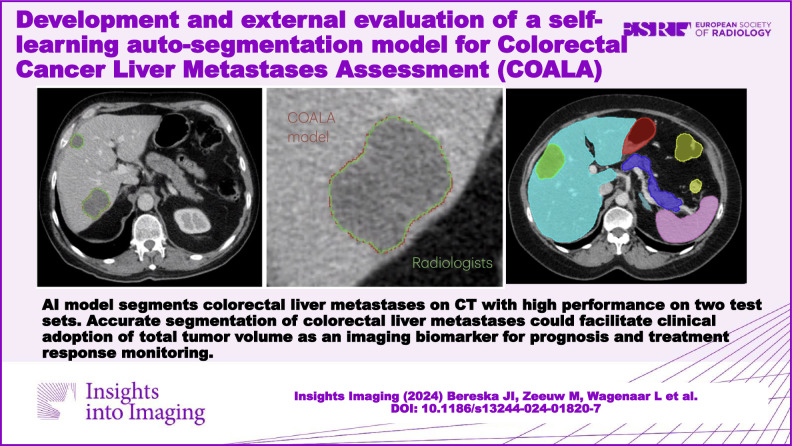

## Introduction

Total tumor volume (TTV) at baseline and TTV response to systemic therapy are prognostic for overall and recurrence-free survival in patients with colorectal cancer liver metastases (CRLM) [1–5]. Currently, the evaluation of response to systemic therapy of CRLM is performed using the Response Evaluation Criteria in Solid Tumors (RECIST1.1) [6]. However, the correlation between RECIST1.1 and survival remains indeterminate [7]. Using TTV might lead to more clinically relevant assessments when evaluating the response to systemic therapy of CRLM. Assessing TTV involves manual segmentation of numerous CRLMs, which is a time-consuming task requiring considerable expertise. Moreover, manual segmentation is subjective, leading to inter-observer variability. Thus, despite its potential prognostic value, TTV assessment has not been adopted in clinical practice. Artificial intelligence (AI) CRLM segmentation models may aid clinicians in automatically assessing TTV, facilitating practical application in routine patient care.

Automatic segmentation of the liver and primary liver tumors has been extensively studied in recent years, with various deep learning architectures such as convolutional neural networks, UNet and UNet variants, and generative adversarial networks being employed to segment primary liver tumors like hepatocellular carcinoma with promising results [8–17]. However, this work focuses on CRLM, which presents unique challenges compared to primary liver tumors due to their heterogeneous appearance and less well-defined borders. Although some work has been done on automatic segmentation of CRLM, it is limited compared to the body of research on primary liver tumors. For instance, Vorontsov et al proposed a semi-automatic segmentation method for CRLM, improving segmentation speed compared to manual segmentations but lacking volumetric accuracy [17]. Similarly, Wesdorp et al introduced an automatic segmentation model for CRLM; however, this model fell short in an external test cohort [16]. This lack of segmentation accuracy underlines the imperative for developing more precise models capable of clinical-grade CRLM segmentation to facilitate automated TTV assessments.

To address current limitations in spatial accuracy of automated CRLM segmentation, we developed a self-learning-based segmentation model for COlorectal CAncer Liver metastasis Assessment (COALA) using a large patient cohort. The COALA model leverages the *teacher-student* dynamic, with a *teacher* model trained on a smaller segmented dataset guiding a *student* model learning from a larger unsegmented dataset. By using averaged ground-truth segmentations consolidated from multiple radiologists, we aim to minimize observer-dependent variations and improve the feasibility of employing TTV assessments in clinical practice.

## Materials and methods

This study retrospectively included data from two medical centers. The Medical Ethics Review Committee of the Amsterdam UMC, the Regional Ethical Committee of Norway, and the Data Protection Officer of Oslo University Hospital approved this study protocol. All patients were managed per institutional practices. All patients signed a written informed consent form permitting the use of their data for studies.

### Datasets

We utilized two datasets for this retrospective study: the *INTERNAL* and *EXTERNAL* datasets. The *INTERNAL* dataset included 783 portal venous phase CT scans (CT-PVPs) from 373 patients registered in the CAIRO5 trial (NCT02162563), a multicenter randomized controlled trial conducted by the Dutch Colorectal Cancer Group between November 2014 and January 2022 in 47 hospitals [18]. The CAIRO5 trial evaluated the optimal systemic induction therapy for patients with initially unresectable liver-only CRLM. The patients were randomized between systemic therapy combinations depending on the primary tumor site and genetic mutation status. These treatment regimens included doublet or triplet chemotherapy in combination with targeted therapy.

The *EXTERNAL* dataset included 50 CT-PVPs from 35 patients enrolled in the Oslo-COMET trial (NCT01516710), a single-center, randomized superiority trial conducted at the Oslo University Hospital between February 2012 and January 2016. The patients were randomly assigned to undergo laparoscopic or open parenchyma-sparing liver resection [19].

Both datasets consisted of CT-PVPs at baseline before systemic therapy and at follow-up after systemic therapy. We collected information on age, sex, systemic induction therapies, and the number of CRLMs (Table 1). The CT acquisition and reconstruction parameters are detailed in Table S1 in the Supplemental Digital Content.

**Table 1:** Patient demographics for the INTERNAL and EXTERNAL datasets

Characteristics	INTERNAL dataset *N* = 373	EXTERNAL dataset *N* = 35
Sex, *n* (%), female, male	136 (36%), 237 (64%)	19 (54%), 16 (46%)
Average age at diagnosis, years (dev)	62 (10.2)	64 (9)
Average number of CRLM at diagnosis, *n* (dev)	12 (15.6)	1 (1)
Average largest CRLM diameter mm (dev)	44 (32)	19.5 (11.1)
Pre-NAT scans, *n* (%)	373 (48%)	31 (62%)
Post-NAT scans, *n* (%)	410 (52%)	19 (38%)

*CRLM* colorectal cancer liver metastasis, *n* number, *dev* standard deviation, *NAT* neoadjuvant therapy

### Data preparation

For the *INTERNAL* dataset, two research team members (M.Z., N.W.) semi-automatically segmented a selection of 120 CT-PVPs from 55 patients with 1113 CRLM using the Tumor Tracking Modality feature of IntelliSpace Portal 9.0® (Philips). Initially, IntelliSpace Portal automatically generated a region of interest based on differences in density. The two research team members manually refined these outlines slice-by-slice for precise segmentation. Three specialist abdominal radiologists (J.H.v.W.: 18 years’ experience, J.v.d.B.: 10 years’ experience, I.N.: 2 years’ experience) independently reviewed and adjusted these segmentations as necessary using the IntelliSpace Portal.

For the *EXTERNAL* dataset, two members of the research team independently performed initial segmentations of 50 CT-PVPs from 35 patients with 72 CRLM using 3DSlicer (5.4.0). Three specialist abdominal radiologists (G.K.:12 years’ experience, T.S.: 15 years’ experience, F.K.K.: 10 years’ experience) each subsequently independently reviewed and, if needed, corrected all these segmentations using 3DSlicer (5.4.0) and MedSeg (1.0).

The corrected segmentations from the three radiologists in the *INTERNAL* and *EXTERNAL* datasets were merged into one single segmentation through the Simultaneous Truth and Performance Level Estimation algorithm (STAPLE) algorithm, henceforth referred to as the merged segmentations [20].

**Fig. 1:**
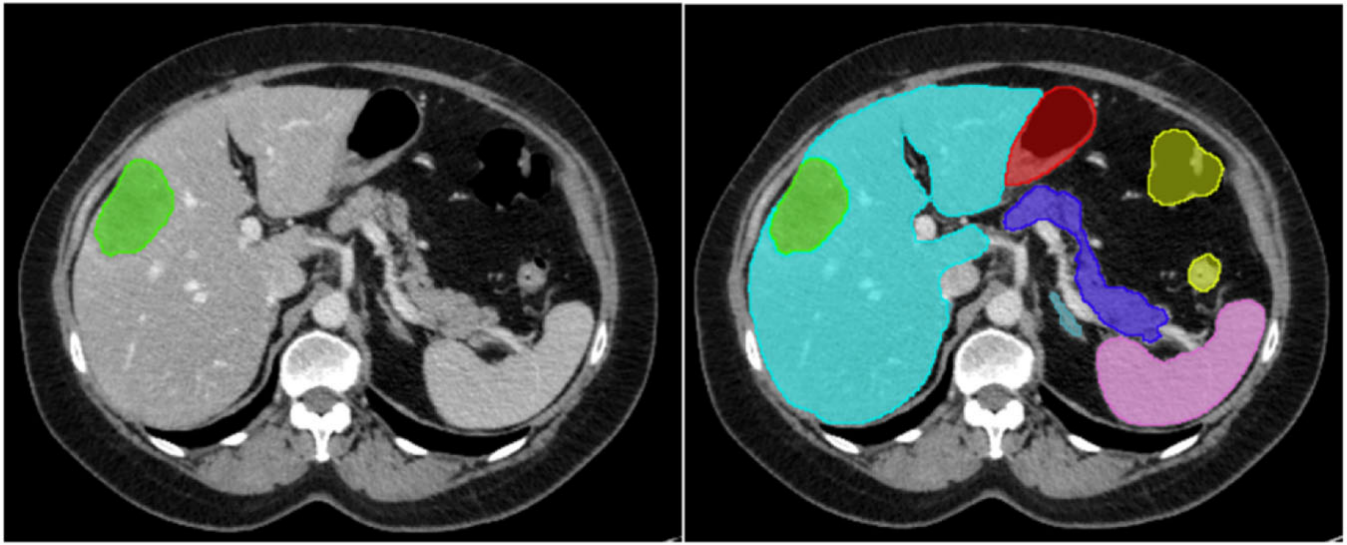
Example of a manually segmented portal venous phase axial computed tomography scan performed by a trio of radiologists and combined using the STAPLE algorithm. Green = CRLM, turquoise = liver, pink = spleen, dark blue = pancreas, light blue = adrenal glands, red = stomach, yellow = colon

Including surrounding abdominal structures has been shown to increase segmentation model performance; therefore, next to the CRLM segmentations, we included segmentations of twelve pertinent surrounding anatomical structures: the duodenum, pancreas, both adrenal glands, spleen, gallbladder, both kidneys, colon, stomach, small bowel, and liver [21]. These additional segmentations were generated automatically using the anatomical segmentation model, TotalSegmentator, and serve as contextual information for the model, helping it to identify relevant areas of the scan and improve CRLM segmentation accuracy [22]. Figure 1 depicts an example of a segmented CT-PVP from the *INTERNAL* dataset.

### Model implementation

**Fig. 2:**
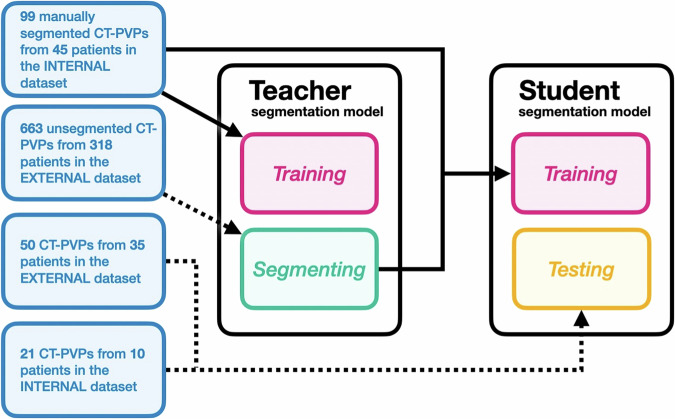
The proposed learning framework for CRLM and abdominal organ segmentation on contrast-enhanced CT scans. CT-PVP, portal venous phase computed tomography scan

We followed a *self-learning* approach to train the COALA segmentation model, which is demonstrated schematically in Fig. 2. Self-learning commences with a *teacher* segmentation model trained on a small set of manually segmented training data. This *teacher* segmentation model is then used to generate segmentations for the entire unsegmented training dataset. These teacher-generated segmentations are subsequently used to train a *student* segmentation model. The *student* segmentation model, through leveraging the additional training data, can exceed the performance of the initial *teacher* segmentation model. This approach can facilitate a reduction in manual segmentations and an increase in the robustness and generalizability of the segmentation model [23].

We initially trained a teacher segmentation model using a subset of 99 CT-PVPs from the previously manually segmented 120 CT-PVPs from the INTERNAL dataset. Using this *teacher* segmentation model, we obtained automatic segmentations of the remaining *INTERNAL* dataset, comprising 663 CT scans from 318 patients. The resulting automatic and initial 99 segmentations were used to train the *student* segmentation model. The *student* model served as the final COALA segmentation model.

We selected a nnUNet network setup that included a two-stage 3D U-Net cascade for both the *student* and *teacher* segmentation models [24]. The cascade comprised an initial U-Net trained on down-sampled images to generate low-resolution segmentations, which served as an auxiliary input for training the subsequent full-resolution U-Net. We used 5-fold cross-validation with an 80:20 training-validation split, 1000 steps per fold, and an initial learning rate of 0.05 to train both the low-resolution and full-resolution U-Nets. All models were trained on an NVIDIA A100 GPU, taking roughly one day per fold.

### Performance assessment

We assessed the performance of the trained COALA model using the 50 CT-PVPs from the *EXTERNAL* dataset and a subset of the *INTERNAL* dataset containing 21 CT-PVPs. To evaluate the spatial accuracy of our model’s CRLM segmentations, we compared the model’s segmentations to the merged segmentations using DICE scores. To evaluate our model’s TTV assessment, we derived the TTV in voxels from the model’s and merged segmentations. We examined the agreement between the model’s and the merged segmentation’s TTV by calculating a two-way mixed-effect intraclass correlation coefficient (ICC), categorizing the results as poor (ICC < 0.4), fair (ICC 0.4–0.59), good (ICC 0.6–0.74), or excellent (ICC 0.75–1.0). Finally, we used Welch’s *t*-test to compare the model’s performance on pre-NAT and post-NAT scans. A *p*-value less than 0.05 denoted statistical significance.

## Results

### Patient characteristics

The INTERNAL dataset contained 783 CT-PVPs from 373 patients, depicting 14,152 CRLM, and the *EXTERNAL* dataset contained 50 CT-PVPs from 35 patients depicting 72 CRLM. In the *INTERNAL* dataset, the majority of patients were male (64%), in the *EXTERNAL* dataset less than half of patients was male (46%). The median number of CRLM at diagnosis (12 versus 1) and the median largest diameter (42 mm versus 19.5 mm) were higher in the *INTERNAL* dataset. Imaging data consisted of CT-PVPs at baseline before systemic therapy (INTERNAL: 373 (48%), EXTERNAL: 410 (52%)) and at follow-up after systemic therapy (INTERNAL: 31 (62%), EXTERNAL: 19 (38%)). See Table 1.

### Segmentation model

**Fig. 3:**
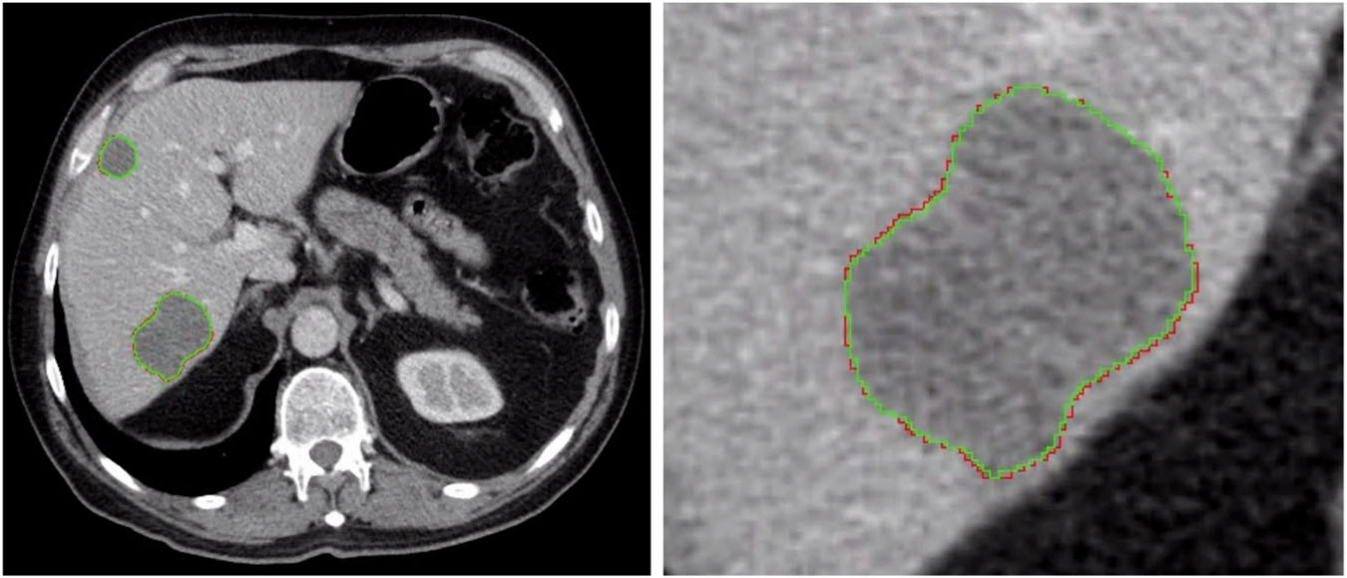
Comparison between the COALA model’s segmentation and the merged segmentation within a portal venous phase axial computed tomography scan. Red = automatic segmentation performed by our model, green = merged manual segmentation performed by three radiologists and merged using the STAPLE algorithm

The COALA model achieved a mean DICE score of 0.83 (IQR: 0.10) on the *EXTERNAL* dataset, with a mean DICE score of 0.84 (0.10) and 0.82 (IQR: 0.05) on pre- and post-NAT scans, respectively. On the withheld subset of the *INTERNAL* dataset, the COALA model achieved a mean DICE score of 0.85 (IQR: 0.05) with a mean DICE score of 0.87 (IQR: 0.02) and 0.85 (IQR: 0.05) on pre- and post-NAT scans, respectively. A Welch’s t-test revealed no significant difference between the model’s performance on pre- or post-NAT scans on either the *EXTERNAL* of the *INTERNAL* dataset (*p* = 0.64 and *p* = 0.22). A visual comparison between the segmentation results garnered by the model and the ground-truth merged segmentation is depicted in Fig. 3.

### Total tumor volume analysis

The agreement of the TTV derived from the COALA model’s with the volumes from the merged segmentations reached an ICC of 0.97 on both the *EXTERNAL* dataset *INTERNAL* datasets. The median, largest, and smallest TTV in voxels obtained from the model’s and the merged segmentations are denoted in Table 2.

**Table 2:** TTV derived from the COALA model’s and the merged segmentations in voxels on the INTERNAL and EXTERNAL datasets

TTV in voxels	EXTERNAL COALA model	EXTERNAL merged	INTERNAL COALA model	INTERNAL merged
Median (IQR)	2,024 (2,573)	2,315 (3,011)	59,760 (135,198)	62,772 (127,396)
Largest	11,240	12,546	656,040	684,647
Smallest	189	122	721	2601

*TTV* total tumor volume, *IQR* interquartile range

## Discussion

This study presents the development and external evaluation of a fully automatic CRLM segmentation and TTV assessment model COALA. By employing a self-learning training setup with a diverse dataset and consolidating CRLM segmentations from three radiologists into a unified ground truth, we reduced the required manual training samples, enhanced the model’s robustness and generalizability, and mitigated observer-dependent variations. The COALA model showed no significant difference in CRLM segmentation DICE scores and displayed near-perfect agreement for TTV assessment in the external evaluation cohort from the Oslo University Hospital. Collectively, these findings suggest that the proposed COALA model has the potential to provide reliable and consistent TTV assessments in routine clinical practice.

While automatic segmentation of primary liver tumors has been extensively studied using various deep learning architectures [8–17], the segmentation of colorectal liver metastases (CRLM) presents unique challenges due to their heterogeneous appearance and less well-defined borders. Vorontsov et al made significant contributions in applying deep learning to TTV assessment for CRLM [17]. Their methodology, based on fully convolutional networks, did offer improvements in segmentation speed but was compromised by a lack of segmentation and volumetric accuracy. Specifically, the DICE score achieved by their automated and even user-corrected CRLM segmentation model was substantially lower than the DICE score achieved by our COALA model (with 0.68 compared to 0.85). Similarly, Wesdorp et al introduced an automatic segmentation model for CRLM, but it fell short in an external test cohort [16]. These limitations underscore the need for more precise models capable of clinical-grade CRLM segmentation. By utilizing a larger and more diverse training dataset of 833 scans, compared to 115 in the previous study, we sought to enhance the model’s ability to generalize to new, unseen data from various patient populations. Furthermore, we strengthened the reliability of our ground truth by incorporating annotations from three experienced radiologists, reducing the risk of individual bias or errors. Lastly, our study included an external evaluation of the COALA model using data from Oslo University Hospital, demonstrating its applicability and effectiveness across different medical centers.

There are several limitations of our study that should be acknowledged. First, the retrospective nature of our study limits the prediction of the model’s efficacy in prospective clinical settings. Second, the merged segmentations were created by radiologists correcting an existing pre-segmentation, likely resulting in higher inter-rater DICE scores and ICC compared to from-scratch segmentations. Third, the external test cohort differed from the training cohort, specifically in the number of CRLMs per patient. While the model’s good performance despite this discrepancy can be considered a strength, it also poses questions about how representative the training data is for a wide range of clinical scenarios. Finally, we did not evaluate our model on a publicly available benchmark dataset, as existing ones, such as the Liver Tumor Segmentation (LiTS) dataset, mainly comprise primary liver tumors, not CRLM [25]. To address this, we make our internal test set publicly available along with our model for future benchmarking. Future studies should incorporate data from global centers and include more clinically representative test cohorts. Automating manual radiological evaluations, such as response evaluation currently done in clinical practice using RECIST1.1 criteria, presents a promising application.

In conclusion, our study introduces the first fully automatic CRLM segmentation model COALA, which aligns with inter-observer agreement for segmentation and displays near-perfect agreement for TTV assessment. The model’s robustness is highlighted by its external evaluation of data annotated by three radiologists, offering a substantial mitigation of observer-dependent variations. These advancements provide a promising foundation for reliable and consistent TTV measurements, crucial for the effective management of patients with colorectal cancer liver metastases.

## Supplementary information

ELECTRONIC SUPPLEMENTARY MATERIAL

## Data Availability

The authors confirm that the data supporting the findings of this study are available within the article and its supplementary material. The documented code, fully trained COALA model, and test set will be made available on GitHub upon publication.

## Abbreviations

AI: Artificial intelligence
COALA: COlorectal CAncer Liver metastasis Assessment
CRLM: Colorectal cancer liver metastasis
CT-PVP: Portal venous phase CT scan
ICC: Intraclass correlation coefficient
RECIST: Response Evaluation Criteria in Solid Tumors
STAPLE: Simultaneous Truth and Performance Level Estimation
TTV: Total tumor volume
