# Supplementary material for: Development and external evaluation of a self-learning auto-segmentation model for Colorectal Cancer Liver Metastases Assessment (COALA)
Source: Insights Imaging. 2024 Nov 22;15:279. doi: 10.1186/s13244-024-01820-7 (PMC11584830; doi:10.1186/s13244-024-01820-7)
Supplement: Supplementary file 1 — ELECTRONIC SUPPLEMENTARY MATERIAL [file 13244_2024_1820_MOESM1_ESM.pdf]

Development and External Evaluation of a Self-Learning Auto-Segmentation Model for Colorectal Cancer Liver Metastases Assessment (COALA)

ELECTRONIC SUPPLEMENTARY MATERIAL

Table S1: Reconstruction and acquisition parameters used to obtain the CTs.

**Parameter**	**INTERNAL dataset**<br>**N = 373**	**EXTERNAL dataset**<br>**N = 50**
Pitch/table speed	0.6 – 1.48 / 19.2 – 182.8 mm	0.6 – 1.08 / 23 – 86.3 mm
X-Ray tube current	157 – 581 mA	90 – 553 mA
kVp	90 – 120 kVp	100 – 120 kVp
Tube rotation speed	0.4 – 0.75 ms	0.5 – 0.8 ms
Slice thickness	3.0 – 5.0 mm	3.0 mm
Width X height	512 – 1024 X 512 – 1024	512 X 512
